# Tubular Overexpression of Gremlin Induces Renal Damage Susceptibility in Mice

**DOI:** 10.1371/journal.pone.0101879

**Published:** 2014-07-18

**Authors:** Alejandra Droguett, Paola Krall, M. Eugenia Burgos, Graciela Valderrama, Daniel Carpio, Leopoldo Ardiles, Raquel Rodriguez-Diez, Bredford Kerr, Katherina Walz, Marta Ruiz-Ortega, Jesus Egido, Sergio Mezzano

**Affiliations:** 1 Division Nephrology, School of Medicine, Universidad Austral de Chile, Valdivia, Chile; 2 Centro de Estudios Científicos, Valdivia, Chile; 3 Hystopathology Division, School of Medicine, Universidad Austral de Chile, Valdivia, Chile; 4 Cellular Biology in Renal Diseases Laboratory, Universidad Autónoma Madrid, Madrid, Spain; INSERM, France

## Abstract

A growing number of patients are recognized worldwide to have chronic kidney disease. Glomerular and interstitial fibrosis are hallmarks of renal progression. However, fibrosis of the kidney remains an unresolved challenge, and its molecular mechanisms are still not fully understood. Gremlin is an embryogenic gene that has been shown to play a key role in nephrogenesis, and its expression is generally low in the normal adult kidney. However, gremlin expression is elevated in many human renal diseases, including diabetic nephropathy, pauci-immune glomerulonephritis and chronic allograft nephropathy. Several studies have proposed that gremlin may be involved in renal damage by acting as a downstream mediator of TGF-β. To examine the *in vivo* role of gremlin in kidney pathophysiology, we generated seven viable transgenic mouse lines expressing human gremlin (GREM1) specifically in renal proximal tubular epithelial cells under the control of an androgen-regulated promoter. These lines demonstrated 1.2- to 200-fold increased GREM1 expression. GREM1 transgenic mice presented a normal phenotype and were without proteinuria and renal function involvement. In response to the acute renal damage cause by folic acid nephrotoxicity, tubule-specific GREM1 transgenic mice developed increased proteinuria after 7 and 14 days compared with wild-type treated mice. At 14 days tubular lesions, such as dilatation, epithelium flattening and hyaline casts, with interstitial cell infiltration and mild fibrosis were significantly more prominent in transgenic mice than wild-type mice. Tubular GREM1 overexpression was correlated with the renal upregulation of profibrotic factors, such as TGF-β and αSMA, and with increased numbers of monocytes/macrophages and lymphocytes compared to wild-type mice. Taken together, our results suggest that GREM1-overexpressing mice have an increased susceptibility to renal damage, supporting the involvement of gremlin in renal damage progression. This transgenic mouse model could be used as a new tool for enhancing the knowledge of renal disease progression.

## Introduction

Kidney disease and renal failure are worldwide health problems and increasing research efforts are required to understand the molecular mechanisms underlying kidney injury to identify new therapeutic approaches.

Gremlin is a highly conserved secreted protein that is present both on the external cell surface and within the ER-Golgi compartment of different cell types [1], [2], affecting diverse biological processes such as growth, differentiation and development [2]. At early stages of development, gremlin is expressed in the mesoderm and inhibits BMP (bone morphogenetic protein) signaling by binding to and blocking BMP activity [3]. Thus, gremlin is considered a BMP antagonist. The *Grem1*-null mouse was the first *in vivo* gremlin model. This model was neonatally lethal, and the embryos presented kidney and lung defects [4]. Gremlin is critical in nephrogenesis but is quiescent after birth and absent in the normal adult kidney [5]. The gremlin gene may be induced in human mesangial cell cultures that are exposed to high glucose levels and is expressed in the kidneys of diabetic rats [6]–[8]. In biopsies obtained from patients with diabetic nephropathy, we have observed gremlin expression in areas with tubule-interstitial fibrosis, and it colocalizes with transforming growth factor-β (TGF-β) [9]. Moreover, gremlin is also expressed in cellular glomerular crescents and in the tubular and infiltrating interstitial cells of human biopsies of pauci-immune glomerulonephritis and chronic allograft nephropathy, broadening the range of activity to a more global role for gremlin in renal diseases [10], [11]. We have recently shown that recombinant gremlin directly regulates profibrotic events in cultured tubulo-interstitial cells and acts as a mediator of TGF-β responses [12]. Blockade of gremlin in experimental models of diabetes, using heterozygous *grem1* mice or gene silencing, has been shown to ameliorate renal damage, including proteinuria and fibrosis, suggesting that gremlin contributes to renal damage progression [13], [14]. Furthermore, gremlin overexpression in rat lungs results in a partly reversible lung fibrosis through the activation of alveolar epithelial cell proliferation [15]. Interestingly, there are no data regarding the direct effect of gremlin in the kidney.

With the interest to investigate the potential role of gremlin in the kidney in physiological and pathological conditions *in vivo*, we generated viable transgenic (TG) mice expressing the human gremlin gene (GREM1) in the renal proximal tubular cells under the control of a specific kidney androgen-regulated promoter (KAP) that can be used as a molecular “on-off” switch.

We found that GREM1-expressing mice presented normal renal function, but they were more susceptible to developing renal damage induced by folic acid (FA) administration (a known experimental model of acute renal injury), suggesting that gremlin plays a pathogenic role in renal damage. These mice could be used as an *in vivo* experimental model to study the role of gremlin in renal diseases, such as diabetic nephropathy.

## Materials and Methods

### GREM1 cloning

Because human and murine mRNAs and proteins for gremlin exhibit high homology (89% and 98%, respectively), all of the experiments were performed using the human sequence. GREM1 cDNA was purchased from the Mammalian Gene NIH Collection (Bethesda, Maryland USA). To facilitate the detection of human gremlin (as opposed to the endogenous mouse protein), we added a c-myc tag to the 3′ portion of GREM1 using PCR with the forward primer 5′AGTGCGGCGGCTGAGGACCC GCCGCACTGACAT-3′ and the reverse primer 5′-ATAGCCGCCGCTTACAGATCCTCTTCTGAGATGAGTTTTTGTTCATCCAAATCGATGGATATGC-3′. To add another signal to the transgene to facilitate detection in transfected cells, we inserted an e-GFP sequence downstream of human gremlin as follows. The IRES-eGFP sequence was obtained by PCR using a pIRES2-EGFP plasmid (Clontech Mountain View, CA, USA) as the template with the following primers: IRES-eGFP-F (5′-TACATTAATGGGCCCGGGATCCGCCCCTC-3′) and IRES-eGFP-R (5′-GGCCATATGCGCCTTAAGATACATTGATG-3′). The GREM1-c-myc and IRES-eGFP fragments were independently cloned into a pGEMT-Easy vector and then sequenced (Macrogen, Seoul, Korea) to confirm the modifications and absence of additional mutations. Next, both the GREM1-c-myc and IRES-eGFP fragments were subcloned into a modified pCDNA3 vector using the *Eco*RI and *Not*I restriction sites, respectively.

### Transfection of EBNA293 and HK-2 cells

To determine whether the pCDNA3-GREM1-myc-IRES-eGFP generated stable proteins, EBNA293 and human renal proximal tubulo-epithelial (HK2 cell line, ATCC CRL-2190, Virginia, USA) cells were both grown in RPMI with 10% fetal bovine serum (FBS), 1% non-essential amino acids, 100 U/ml penicillin, 100 µg/ml streptomycin, Insulin Transferrin Selenium (5 µg/ml) and hydrocortisone (36 ng/ml) in 5% CO_2_ at 37°C and were then transfected. EBNA293 cells were transfected with 0.6 µg of plasmid using LF2000 (Invitrogen, Carlsbad, CA, USA). Immunofluorescence was performed using a mouse anti-c-myc antibody (M4439, clone 9E10, Sigma, St. Louis, USA) at a 1/500 dilution followed by a donkey anti-mouse IgG conjugated to Cy3 antibody (Jackson ImmunoResearch, West Grove, PA, USA) at a 1/800 dilution. Images were captured using a Zeiss Axiovert 100M epifluorescence microscope. HK2 cells were grown in the aforementioned conditions and at 60–70% of confluence; cells were growth-arrested in serum-free medium for 24 hours before experiments. HK2 cells were transiently transfected for 48 hours using FuGENE (Roche, Basel Switzerland) and the pCDNA3-GREM1-c-myc-IRES2-eGFP plasmid vector. To study the expression of GREM1 in renal-specific tubulo-epithelial cells, HK2 cells were transiently transfected with pKAP-GREM1-c-myc-IRES2-eGFP to confirm the promoter activity. Immunocytochemical studies were performed in cells grown on coverslips. The cells were then fixed in Merckofix (Merck, Darmstadt, Germany) and permeabilized with 0.2% Triton-X100 for 1 min. After blocking with 4% bovine serum albumin and 8% of the corresponding serum (secondary antibody) for 1 hour, the cells were incubated with primary antibodies overnight at 4°C, followed by an Alexa Fluor 633-conjugated antibody (Invitrogen) at a 1/300 dilution for 1 hour. Negative control samples were processed in the absence of primary antibody. The cells were mounted in Mowiol 40–88 (Sigma) and examined using a Leica DM-IRB confocal microscope.

### Transgenesis, genotyping and colony expansion

The pKAP plasmid, which has been previously shown to be specific in males [16]–[20], was used as the backbone for transgene generation. The pKAP plasmid was modified by excising an exon of the human angiotensinogen gene and the poly (A) signal. First, the IRES-eGFP sequence was subcloned downstream of GREM1-c-myc into pGEMT-Easy. The resulting construct was digested with *Sph*I and *Nde*I, and the 2.3 kb fragment containing GREM1-myc IRES-eGFP was subcloned into the modified pKAP plasmid. The 4.1 kb-long transgene was isolated with *Ase*I and *Eco*RV and purified using the QIAEx II kit (QIAGEN, Valencia, CA, USA). Five hundred molecules were microinjected into hybrid C57BL/6J x CBA/J zygotes, which were then transplanted into 13 pseudopregnant mothers. The born mice were maintained in a specific pathogen-free mouse facility in a 12-hour light:dark cycle with access to food and water *ad libitum*. The sacrifice of animals was done with administration of anaesthesia and analgesia, following the protocols approved by the Committee on the Ethics of Animal Experiments of Universidad Austral de Chile (Permit Number:20.2011), and FONDECYT Ethics Committee, and according to the NIH Guidelines. The founders and pups were screened using PCR with the following primers: *GREM1* intron 1F (5′-GCCAGTA AGGAATTCTAATAGG-3′), *KAP* promoter F (5′-ATGAGGACTCTAA TGCGTACAT-3′) and *GREM1* exon 2R (5′-TCCAAATCGATGGATA TGCAAC-3′). The PCR reaction generated two differential products (820 bp endogenous; 1040 bp TG). Once the genotype of the founders was confirmed, the founders were mated with pure C57BL/6J mice to expand the colony. F1 mice were screened using PCR as previously described and used for further molecular and phenotypic characterization studies.

### Mouse characterization

First, kidneys from 4 to 5 week-old mice were analyzed by indirect immunofluorescence using a rabbit anti-GFP antibody at a 1/100 dilution (Invitrogen) and immunohistochemistry (IHC) to detect c-myc. Following confirmation that the female transgenic mice did not express the transgene, these mice were used only for mating and to evaluate the off/on system by the administration of 2.5 mg of testosterone via an intraperitoneal (i.p.) injection over five consecutive days [17], [18]. Male TG and wild-type (WT) mice of five selected lines were also analyzed using western blotting analyses. The kidneys were dissected from both WT and TG mice homogenized in lysis buffer (125 mM Tris pH 6.8 and 1% SDS) supplemented with 1X protease inhibitor cocktail (Sigma P8340). Twenty micrograms of protein was electrophoresed in a 4–12% SDS-PAGE gel and transferred onto a PVDF membrane (Bio-Rad, Dreieich, Germany). The membrane was incubated with anti-c-myc at 1/2500 or anti-actin at 1/5000 (Sigma). Densitometric analysis of the immunoreactive bands was performed using Quantity One software (Bio-Rad).

### mRNA expression

Total RNA was extracted with TRIzol according to the manufactureŕs instructions and quantified using Qubit reagent (Invitrogen). RNA was treated with DNase I (Ambion, Austin TX, USA) to remove potential contamination and reverse transcribed using random primers and the ImProm-II kit (Promega) to synthesize double-stranded cDNA. qPCR was performed with the commercial reagent Maxima SYBR Green qPCR Master Mix (Promega, Madison WI, USA) to determine *GREM1, cyclophilin* and *GAPDH* mRNA expression levels using the following primers: human GREM1 F (5′-CCCGGGGAGGAGGTGCTGGAGT-3′); human GREM1 R (5′-CCGGATGTGCCTGGGGATGTAGAA-3′); mouse cyclophilin1 F (5′-GCAGACATGGTCAACCCCACCG-3′); mouse cyclophilin1 R (5′-GAAATTAGAGTTGTCCACAGTCGG-3′); mouse GAPDH F(5′-TCCGCCCCTTCTGCCGATG-3′); and mouse GAPDH R (5′-CACGGAAGGCCATGGCAGTGA-3′). PCR product specificity was verified by melting curve analysis, and all of the real-time PCR reactions were performed in triplicate. The 2^−ΔΔCT^ method was used to analyze the relative changes in gene expression levels [21].

### Induction of acute renal damage in TG mice

The adult TG lines A and D and wild-type (WT) male littermates aged 4 to 5 months were used. TG and WT mice were injected i.p. with 250 mg/kg body weight of FA (Sigma F7876), dissolved in the vehicle 0.3 M sodium bicarbonate (veh). Control animals, both WT and TG, received 0.3 ml of veh. Additional studies were done in transgenic homozygous mice from line A, injected with FA or vehicle (used as control, because there were not wild type littermates). Spot urine and serum were collected on days 0, 7 and 14 from all of the animals and analyzed for proteinuria and creatininuria using Bradford assay (Bio-Rad) and a Creatinina Wiener Lab Kit (Wiener Laboratorios, Rosario, Argentina), respectively. Seven or 14 days after the injection, the animals were anesthetized with 2% 2,2,2-tribromethanol (Sigma) dissolved in 2-methyl-buthanol (Sigma). The kidneys were removed, decapsulated and cut along the sagittal plane. The left kidney was fixed in 4% formaldehyde, while the right kidney was immediately frozen in liquid nitrogen and processed for RNA and protein extraction. The specimens were embedded in paraffin and cut into 4 µm tissue sections for further histological (PAS/Masson) and IHC studies using antibodies against gremlin and αSMA, F4/80, CD3 and PCNA.

### Histological analysis and IHC

Tubular and interstitial lesions were graded from 0 to 4 and analyzed as previously described [22]. IHC for different markers was performed following heat-induced epitope retrieval (microwaving for 10 min in citrate buffer), and sections were incubated overnight with rabbit anti-human gremlin 1/300 (AP6133a, Abgent, San Diego, CA, USA), followed by incubation with Impress anti-rabbit reagent (Vector, Burlingame, CA, USA); or mouse anti-αSMA 1/100 (DAKO, Carpinteria, CA, USA), monoclonal anti-c-myc clone 9E10 1/300 (Thermo, Rockford IL, USA) or anti-PCNA (PC10, DAKO) followed by incubation with the M.O.M. Immunodetection kit (PK 2200 Vector). All of the tissue sections were developed using AMEC red chromogen (SK 4285, Vector) or DAB, and counterstained with hematoxylin. Interstitial infiltrating cells were detected by mean of F4/80 (monocytes/macrophages) and CD3 (T lymphocytes) antibodies. F4/80 was detected by using the MA1-91124 antibody (dilution: 1/100, THERMO, Rockford, IL, USA) followed by Immpress Reagent Kit (MP 7444, Vector, USA) and CD3 was detected using Trilogy epitope retrieval (Cell Marque, Rocklin, USA) and A 0452 (181–195) antibody (dilution: 1/200, DAKO, USA) followed by horseradish peroxidase streptavidin (dilution 1∶500, SA-5004 Vector, USA), reveled with DAB, and counterstained with hematoxylin.

Murine Gremlin was detected using anti-murine Gremlin antibody (dilution: 1/20 AF 956, R&D Systems, Minneapolis, MN, USA) overnight at 4°C, and the reaction was developed with Impact NovaRed SK-4805 (Vector, USA).

Image analysis and quantification of the IHC signals were performed using the KS300 imaging system, version 3.0 (Zeiss). For each sample, the mean staining area was obtained by an analysis of 20 fields (20x). The staining score is expressed as the mm^2^/dens

### Statistical analysis

The results were expressed as the means ± SEM. Two-tailed chi-square tests were performed to determine the statistical significance of the viability of the transgene and the proportion of female:male pups born in the TG lines. A factorial ANOVA followed by the Tukey test was performed to compare proteinuria and *Grem1* mRNA expression. The Mann-Whitney U-test was performed to compare the tubular/interstitial lesions and Grem1, αSMA, F4/80 and CD3 IHC signals in the TG and WT mice injected with FA. A Spearman rank correlation was performed to determine the correlation between GREM1 and TGF-β, and Kruskal-Wallis analysis was performed to evaluate endogenous murine gremlin and PCNA expression. Values of p< 0.05 were considered significant.

## Results

### Generation of TG GREM1 mice

**Figure 1:**
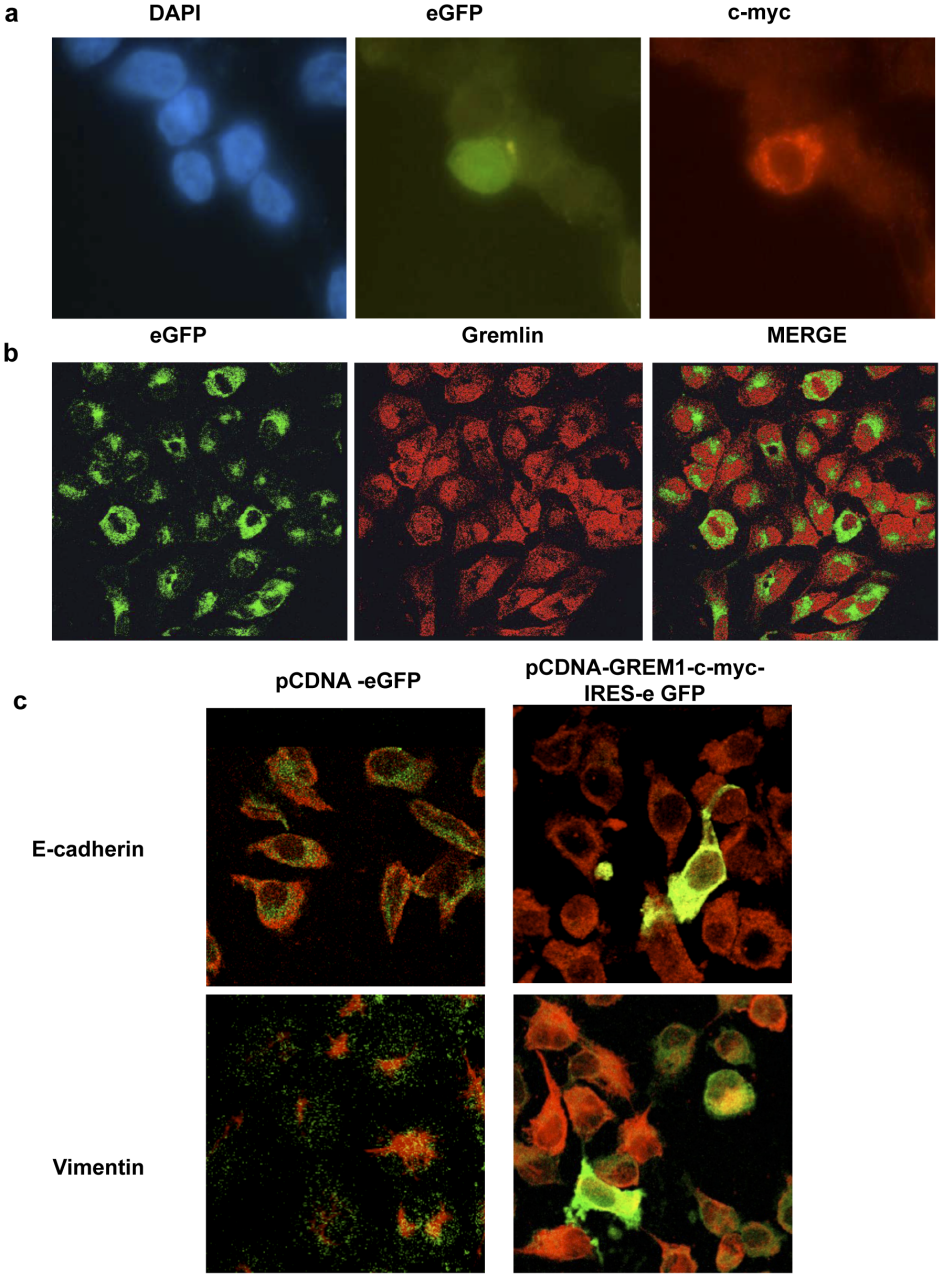
In vitro validation of the GREM1 plasmid. (a) EBNA293 cells were transfected with pCDNA3-GREM1-c-myc-IRES-eGFP, as described in the methods. Immunofluorescence shows that eGFP (green) and c-myc (red) are expressed in the same transfected EBNA293 cell. Nuclei were stained with DAPI (blue) (1000x). (b) In HK-2 cells transfected with pCDNA3-GREM1-c-myc-IRES-eGFP, GREM-1 expression was evaluated by immunocytochemistry using an antibody against GREM-1, followed by a secondary TRICT antibody (red staining). The figure shows eGFP and GREM-1 expressed in the same cell (800x). (c) Confocal immunofluorescence of HK-2 transfected cells, showing the loss of E-cadherin and induction of vimentin in eGFP-positive GREM-1-expressing cells (1600X). E-cadherin and vimentin immunostaining was detected with secondary anti-FITC antibodies (green).

**Validation of the GREM1 expression vector.** Expression of pCDNA3-GREM1-c-myc-IRES-eGFP in EBNA293 cells revealed that this construct produced stable proteins of c-myc-GREM1 and eGFP, and both were expressed simultaneously in these cells (Figure 1a). To distinguish the human TG protein from the murine endogenous gremlin, a c-myc tag was fused to the GREM1 protein, and we used eGFP as reporter for transgene expression. *In vitro* experiments in EBNA293 cells were performed to detect the c-myc signal and showed an expression pattern consistent with GREM1 subcellular localization [1], [2]; in a similar pattern to that found in human renal biopsies [9]–[11], where this protein is localized in cytoplasm and nucleus of the affected tubular epithelial cells. HK-2 cells transfected with pCDNA3-GREM1-c-myc-IRES-eGFP were positive for GREM1 (Figure 1b), which confirmed that eGFP could be used as a reporter for *in vivo* experiments and that c-myc could be used to differentiate human GREM1 in TG animals.

To validate our TG construction, exclude loss of function of the GREM1-c-myc-fused protein and determine whether GREM1 was functional, several experiments were performed in HK2 cells. We have previously demonstrated that stimulation with recombinant GREM1 protein in HK2 cells induces phenotypic changes related to epithelial to mesenchymal transition (EMT) [12]. Transient transfection of these cells with pCDNA3 GREM1-c-myc IRES-eGFP also induced characteristic EMT features, such as the downregulation of E-cadherin immunostaining and an induction of vimentin expression, as well as changes in the cell phenotype to a fibroblast-like morphology, confirming that our expression vector displayed similar effects to the GREM1 recombinant protein (Figure 1c).

Thus, to specifically induce the expression of GREM1 in proximal tubular renal cells, we used the promoter of kidney androgen-regulated protein (pKAP) to drive transgene expression because it is transcriptionally active only in these cells and its activity is testosterone dependent.

**Figure 2:**
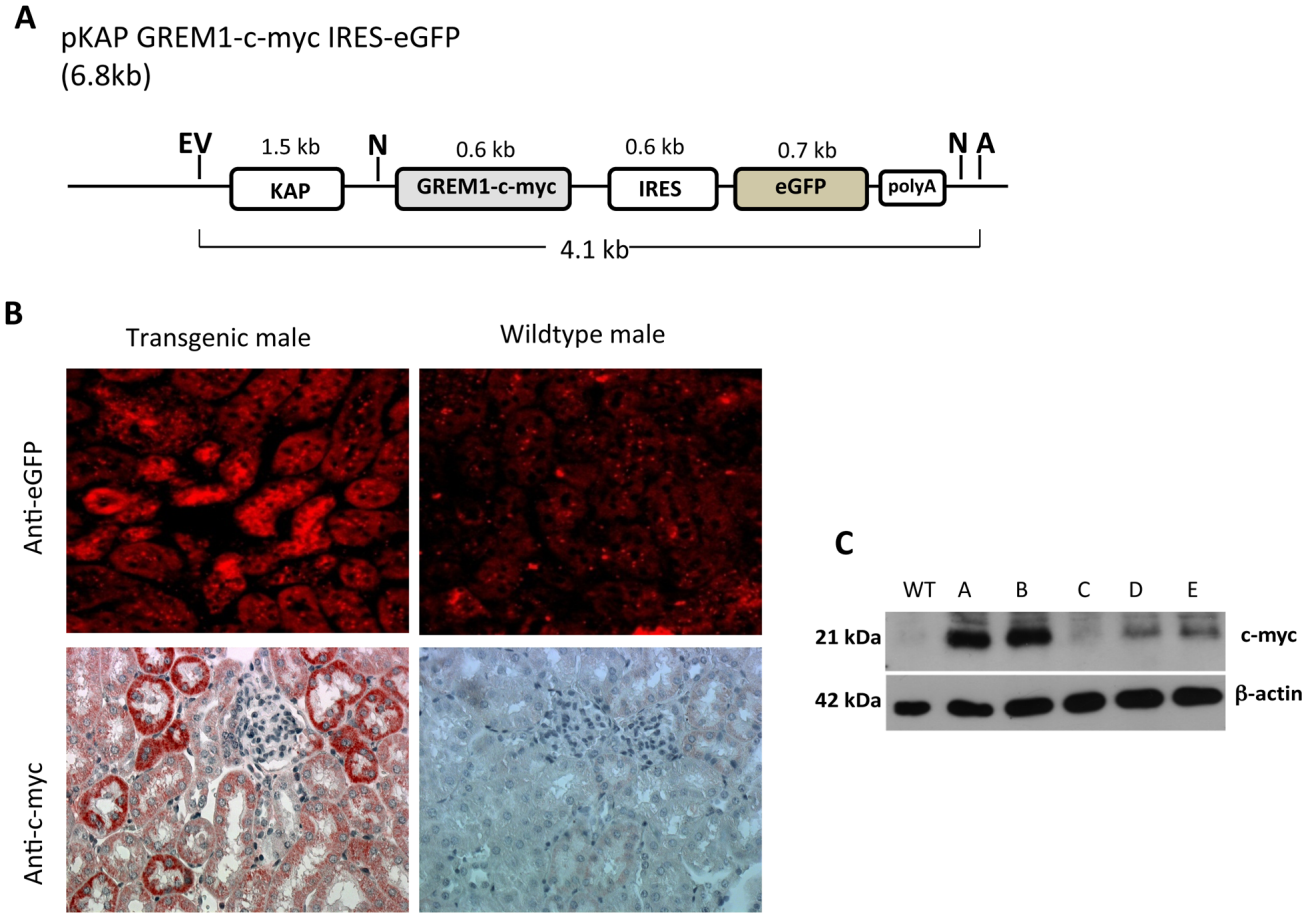
Generation and validation of transgenic mice with specific tubular GREM1 overexpression. (a) Illustration of the pKAP GREM1-c-myc-IRES-eGFP plasmid. Restriction sites used for transgene isolation are indicated with EV (*Eco*RV) and A (*Ase*I). (b) **eGFP and c-myc detection in renal tubular epithelial cells of transgenic mice.** Immunofluorescence against eGFP and immunohistochemistry for c-myc (peroxidase immunostaining) to detect these proteins in the kidney tissue of transgenic males from line A and WT mice (400x). (c) Kidneys were dissected from WT and transgenic male mice of lines A, B, C, D and E, and isolated proteins were subjected to western blotting using an antibody against c-myc (1∶1000); anti β-actin (1∶2500) was used as a loading control. GREM1 expression was determined by densitometric analysis of the c-myc/β-actin ratio and normalized to transgenic line C expression.

Transgene isolation was performed by digestion of the pKAP GREM1-c-myc IRES-eGFP plasmid (Figure 2a) with *Ase*I and *Eco*RV, which generated three fragments of 4.1, 1.5 and 1.2 kb. The 4.1 kb fragment was purified and quantified to obtain 500 molecules/picoliter and then microinjected into C57BL/6J x CBA/J hybrid zygotes. Ninety percent (232/259) of the microinjected zygotes were transferred to pseudopregnant mothers. Eight of the 57 (14%) pups born were confirmed to be TG using PCR. All of the founders reached the age of 3 months, and seven of the founders were subsequently mated with pure C57BL/6 to generate F1 mice.

### Molecular and phenotypic characterization of GREM1 mice

TG lines (named in alphabetic order (A–G)) demonstrated normal fertility and litter sizes. Transgene transmission from all of the lines was verified using PCR analysis and appeared normal in the first and second (F1 and F2) generations in all lines but was significantly increased in line D (p ≤ 0.001) (Table 1). In all TG lines, Mendelian ratios were observed between TG/WT males and females at birth, discarding an effect of transgene expression on male survival during gestation (Table 1).

**Table 1:** Molecular characterization of transgenic mice.

Line	Copy number	Transmittance F1–F2 (%)	eGFP signal (IIF)	GREM1 levels (mRNA)	GREM1 levels (c-myc)	Male/Female (%)	Ectopicity
A	44	54	+++	190 ± 92	7.54	55/45	2
B	2–3	53	+	3.9 ± 0.4	6.38	52/48	1
C	2	50	++	2.2 ± 0.7	1.00	45/55	2
D	8–18	81***	++	10.5 ± 11.1	2.07	53/47	2
E	24	49	++	9.7 ± 7.2	2.4	48/52	1
F	11	46	+	54.6 ± 29.9	n.d	48/52	1

Qualitative and quantitative data from the transgenic lines are shown. Copy number and GREM1 expression levels were determined using real-time PCR. The eGFP signal was qualitatively analyzed. c-myc levels were quantified by densitometric analysis in each line and normalized to line C. Ectopic expression is indicated as the number of the eight analyzed extrarenal organs that demonstrated increased for GREM1 expression compared with wild-type expression. M, male; F, female; n.d., no data. *** p < 0.001.

Males and females were subjected to immunofluorescence analysis for eGFP and immunohistochemistry for c-myc. Males showed specific tubular epithelial expression of eGFP that was qualitatively variable in each TG line (Table 1). The eGFP and c-myc signals were not detected in WT males (Figure 2b). Moreover, this signal was not detected in TG females but could be induced in testosterone-treated TG females (data not shown), verifying the functionality of the promoter as previously described [18], [20].

To confirm that the TG mice expressed a stable TG protein, western blotting analysis for c-myc-tagged GREM1 was performed. The assay detected a protein with the expected molecular weight (∼21 kDa) in the kidneys of the TG lines, and this c-myc-tagged protein was absent in WT mice (Figure 2c). Densitometric analysis revealed that the GREM1 expression levels were variable in each TG line and ranged between 1 (line C exhibited the lowest expression) and 7.5 (line A exhibited the highest expression) (Table 1). Line G was not used in further experiments because the RT-PCR analysis detected ectopic expression of GREM1 in seven extra-renal tissues. The other six TG lines showed low extra-renal expression (cerebellum, data not shown). The levels of GREM1 mRNA in the kidney ranged between a 2- and 200-fold increase compared with the WT mice and were related to the transgene copy number (2 to 44 transgene copy number) (Table 1). Proteinuria (urine protein to creatinine ratio) was monitored in the first generation of males every 2 weeks until the age of 6 months in the three groups (WT and TG lines B and D), and no abnormal increases were observed compared with the WT mice, indicating that GREM1 expression alone was not sufficient to develop renal injury. Moreover, no histological lesions were observed at 6 months of age in any group.

### Renal injury induction in GREM1 TG mice

To determine the effect of GREM1 in renal damage *in vivo*, we selected mice from two lines and challenged these mice with a model of FA-mediated acute renal failure [23].

**Figure 3:**
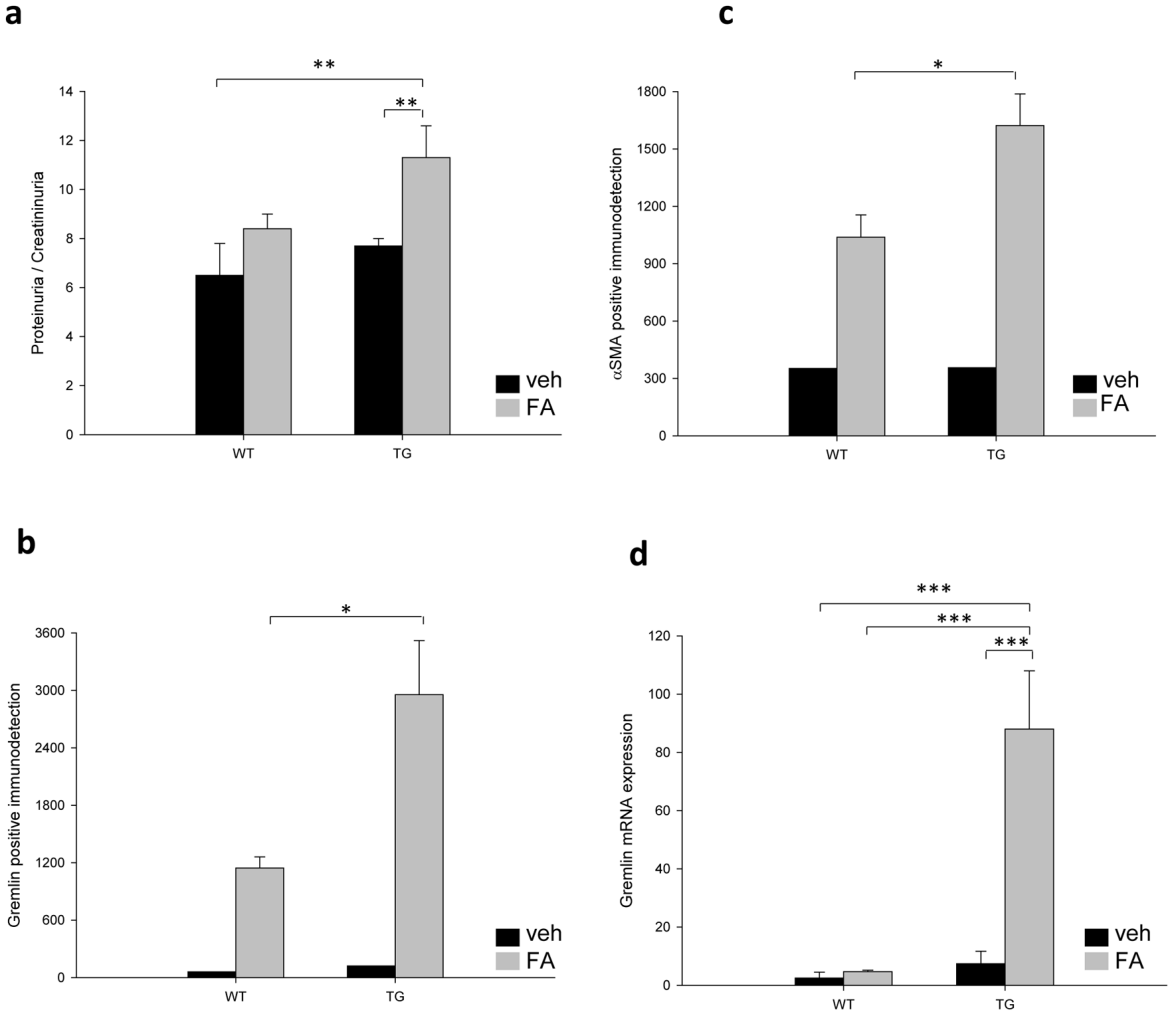
FA injection induces proteinuria and GREM1 and αSMA expression in transgenic line D. (a) The urinary protein to creatinine ratio (µg/mg) was examined in each experimental group. Positive IHC signals were quantified for (b) gremlin and (c) αSMA with KS300 image analyzer software. (d) Gremlin mRNA expression levels were determined using real-time PCR. The four parameters were significantly increased in GREM1-overexpressing transgenic mice. Data are shown as the mean ± SEM of 5-6 mice per group * p < 0.05; ** p < 0.01; *** p < 0.001. TG vs WT control

The line D, TG mice (8–18 transgene copies) were evaluated first. Seven days after FA injection, TG mice presented a significant increase in proteinuria compared with the treated WT mice (Figure 3a). However, the morphological lesions score found at 7 days, did not reach statistical significance between TG-FA compared with WT-FA (TG 9.0 ± 1.0 vs. WT 8.0 ± 2.1; p  =  0.328, n = 5–6 mice per group). To further evaluate profibrotic markers, IHC for αSMA, as the first phenotypic marker of activated fibroblasts, was performed. In FA-TG mice, αSMA was markedly upregulated, showing a significant increase compared with FA-treated WT mice (Figure 3c).

Gremlin expression was also evaluated using real-time PCR. In FA-injected TG mice, (TG-FA) gremlin mRNA expression showed a nearly 10-fold increase compared with veh-injected mice and was significantly higher than FA-treated WT mice (WT-FA) (Figure 3d). Similar findings were observed by IHC, with a significant increase in gremlin staining after FA injection in TG mice compared with WT-FA mice (Figure 3b). Furthermore, we found a significant correlation between αSMA and gremlin IHC signals (F_1,10_: 14.34; p < 0.0356; r  =  0.5892), indicating that gremlin is associated with the variation in αSMA (r  =  0.5892). Taken together, these results indicated that the effect observed *in vitro* [12] could be corroborated *in vivo*.

**Figure 4:**
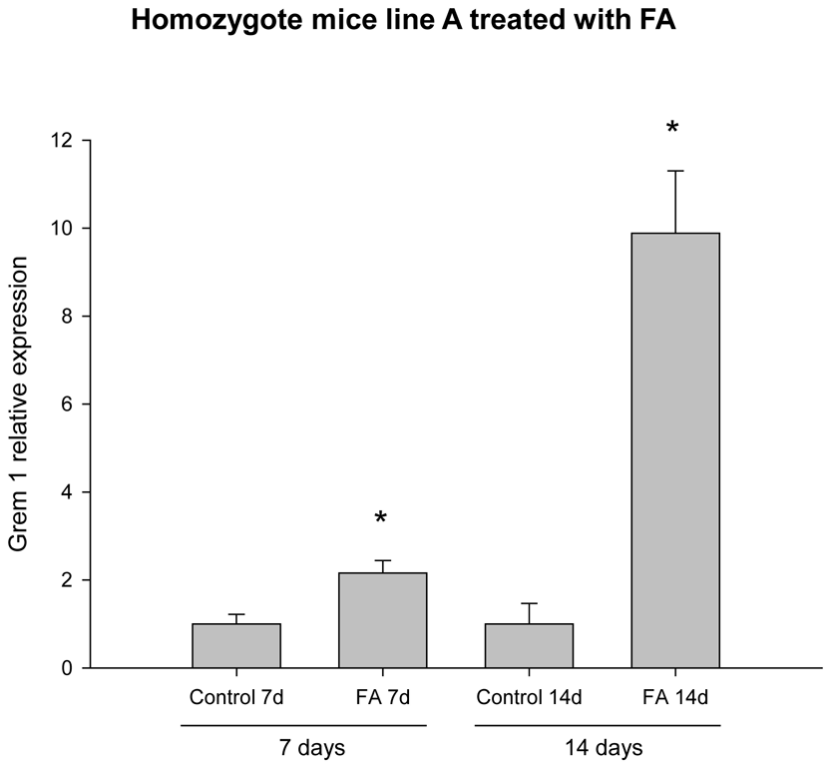
Effect of FA administration on GREM1 expression in transgenic line A homozygous mice. Gremlin expression in transgenic mice was examined 7 and 14 days after treatment with FA. The GREM1 relative expression in homozygous mice of transgenic line A was increased at 7 days after injection with FA and remained increased at 14 days. Data are shown as the mean ± SEM of 4-9 mice per group * p≤0.0049 TG-FA vs TG-Veh, used as control because no WT littermates of the transgenic homozygotes mice were available.

**Figure 5:**
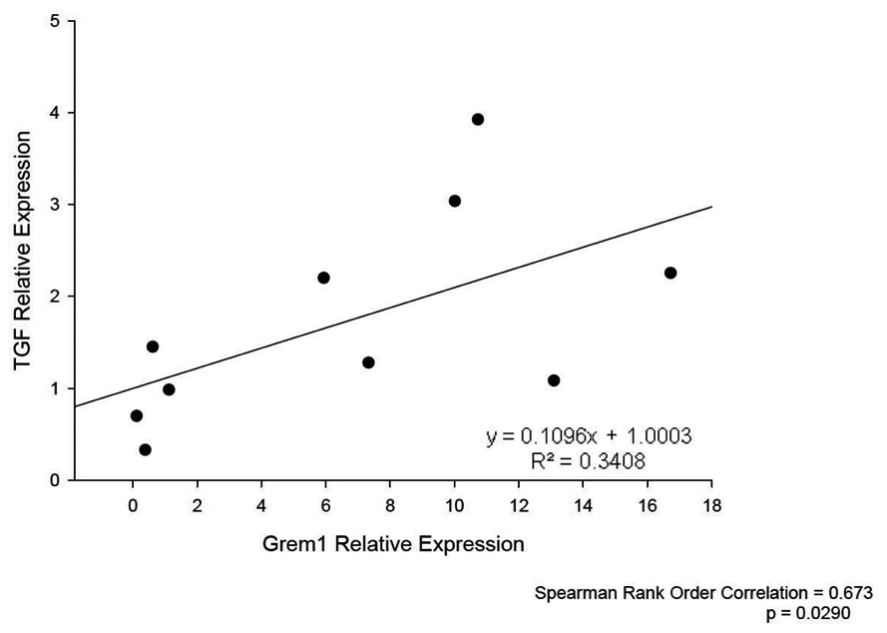
Correlation of TGF-β and GREM1 expression in transgenic line A homozygous mice. TGF-β gene expression was measured in FA-injected GREM1 transgenic mice. We observed a strongly positive correlation between TGF-β and GREM1 expression (p≤0.029, R  =  0.67; 4-9 mice per group).

**Figure 6:**
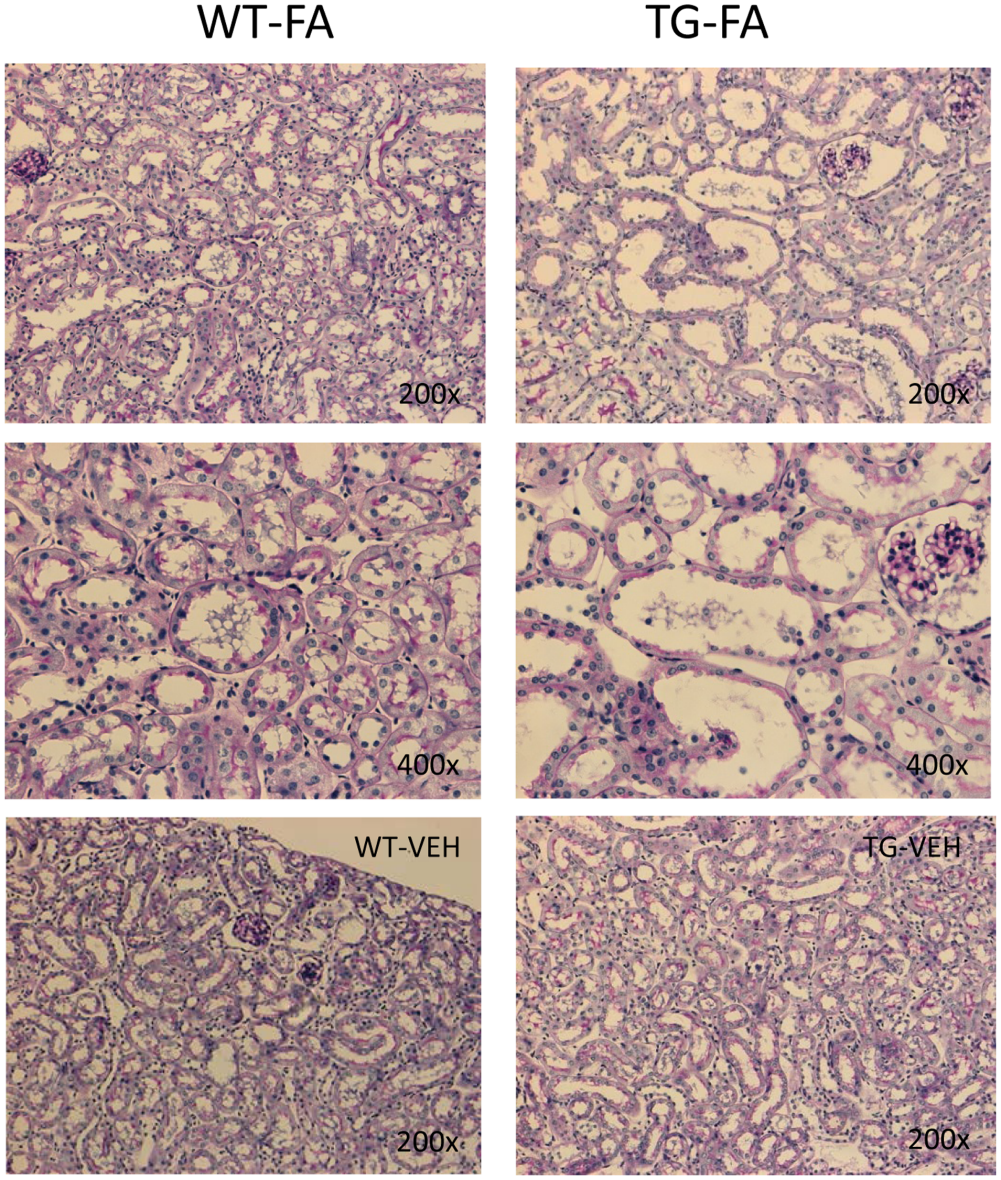
Histological analysis (PAS, Masson) of FA-injected mice in transgenic line A homozygous mice. TG-FA mice (right column) showed more severe morphological lesions (tubular dilatation, flattening of tubular epithelial cells, hyaline casts, interstitial infiltrating cells and mild interstitial fibrosis) compared with WT mice (left column). p < 0.05 (200x-400x). Control vehicle treated mice are shown at bottom. Figure shows representative mice of each group of 14–18 studied.

**Figure 7:**
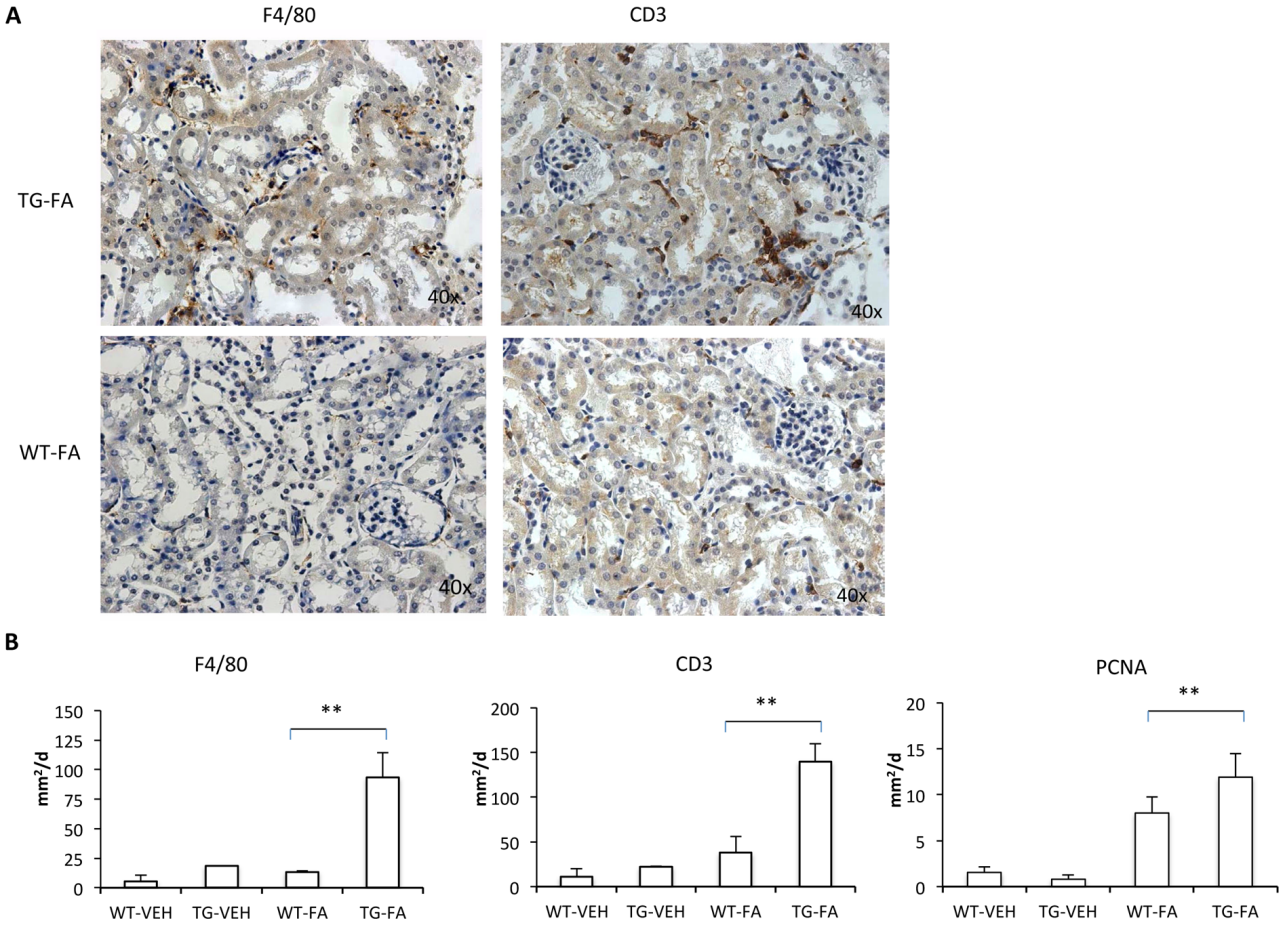
FA injection induces interstitial cell infiltration in transgenic line A homozygous mice. The inflammatory cell infiltration was characterized by immunohistochemistry with anti-F4/80 (monocytes/macrophages) and anti-CD3 (T cells) antibodies. (A and B). Representative immunostaining of one mouse from each group (x400 magnification). (C) Quantification of positive IHC signals were quantified for (a) F4/80, (b) CD3 and (c) PCNA using KS300 image analyzer software. All parameters were significantly increased in transgenic mice. Data are shown as the mean ± SEM of 14–18 mice per group * p < 0.05; ** p < 0.01 vs WT-FA.

To further evaluate the effect of gremlin in FA-induced damage and determine if this was a transient or sustained effect, we performed additional studies using homozygous mice from TG line A and additionally evaluated its expression 14 days after treatment with FA. This line had the greatest number of copies of GREM1 (88 transgene copies) and gremlin mRNA. By real time PCR, we demonstrated that Grem1 was significantly increased at 7 and 14 days after FA injection (Figure 4) and by IHC gremlin renal staining was also increased at 7 and 14 days, with higher level at 14 days after treatment (score, expressed as mean of percentage/mm^2^: TG-VEH; 14, TG-FA at 7 days; 343, TG-FA at 14 days; 862, p ≤ 0.0049), indicating that GREM1 expression in TG mice induces higher renal synthesis of gremlin following FA administration. Additionally, gremlin has been proposed as a downstream mediator of TGF-β [6], and we have previously reported that TGF-β induced gremlin expression in renal tubular cells *in vitro* [12]. Therefore, to further evaluate profibrotic factors, TGF-β gene expression was measured. In FA-injected GREM1 TG mice, an increased relative renal expression of TGF-β at 7 and 14 days after treatment was observed compared with WT mice. Moreover, a strongly positive correlation was found between TGF-β and gremlin expression (p ≤ 0.029, R  =  0.67) (Figure 5). Renal histological analysis at 14 days showed more severe morphological lesions (tubular dilatation, epithelium flattening, hyaline casts, interstitial cell infiltration and mild interstitial fibrosis) in TG-FA mice compared with WT-FA mice (p =  0.0037 Fisher's test, and Mann-Whitney p< 0.01) (Figure 6). To further evaluate inflammatory cell infiltration and proliferation, IHC for F4/80 (murine macrophages), CD3 (murine T cells), and PCNA was performed. In TG-FA mice, all markers were strongly upregulated, showing a significant increase compared with WT-FA mice (Figure 7). Also, a positive correlation was found between gremlin expression and PCNA at 14 days (R  =  0.88, p ≤ 0.0019; data not shown)

## Discussion

We reported here, for the first time, the generation of TG mice expressing GREM1 in a sex- and renal tubular cell-specific manner with no evident lethal effects and normal renal function and morphology. This mouse model was designed as a molecular tool to analyze the effect of gremlin expression in renal damage. Our results suggest that under normal conditions, GREM1 in adult tubular cells is not sufficient to cause renal damage. However, in response to acute renal injury caused by FA injection, GREM1 TG mice presented exacerbated renal damage, suggesting that gremlin could participate in renal damage progression *in vivo*. Gremlin is a developmental gene involved in renal morphogenesis due to its role as a BMP antagonist, but its function in the adult kidney is unknown. Several *in vitro* studies have evaluated the effect of gremlin in renal cells; however, the *in vivo* function has not been investigated. In tubular epithelial cells *in vitro*, overexpression of this expression vector resembles the effect of recombinant GREM1, inducing phenotypic changes related to EMT [12].

These pKAP GREM1-overexpressing mice, which presented a specific GREM1 expression pattern in kidney tubular epithelial cells, demonstrated normal renal function and morphology. The specific pKAP promoter [20], motivated the specific cell type and hormone-regulated targeting of transgene expression. In addition, due to the developmental role of gremlin, expression driven by the pKAP promoter prevented any lethal effects at embryonic stages and generated a testosterone-dependent off/on switch in TG females.

Studies in TG mice overexpressing profibrotic factors, such as connective tissue growth factor (CTGF-CCN2) in different tissues have shown similar findings, as observed in our renal GREM1 TG mice. Although *in vitro* studies have shown that recombinant CCN2 increased extracellular matrix production [24], as observed with gremlin, several *in vivo* studies have shown that CCN2 alone is not sufficient to cause ongoing profibrotic changes. In the kidney, podocyte-specific CCN2-TG mice (in C57BL/6 background) exhibit no glomerular abnormalities, proteinuria or matrix accumulation [25]. In C57BL/6 mice, systemic CCN2 administration has been shown induce a transient overexpression of profibrotic genes at day 5, but it is not sufficient to induce progressive fibrosis [26], as observed following CCN2 overexpression in rat lungs [27]. In a mouse skin model, only coinjection of CCN2 and TGF-β1, not either cytokine alone, caused persistent fibrosis [28].

Several authors have suggested that gremlin could be considered as a mediator of renal injury in diabetic nephropathy, based on experimental studies showing a beneficial effect of gremlin inhibition [13], [14]. In response to FA-induced acute renal damage, GREM1 TG mice developed higher proteinuria after 7 and 14 days than WT mice. Tubular GREM1 overexpression was associated with renal upregulation of profibrotic factors, such as TGF-β and αSMA, recruitment of F4/80 and CD3 positive cells, and increased cell proliferation in TG mice challenged with FA compared with WT mice. Furthermore, as hypothesized these GREM1 overexpressing mice developed more severe histological damage in response to FA injection at 14 days, particularly those mice with more transgenic copies and at the time of more gremlin expression.

In biopsies from patients with diabetic nephropathy, we have demonstrated that GREM1 is expressed in areas of tubular-interstitial fibrosis and that it colocalizes with profibrotic markers, including TGF-β, αSMA and vimentin. These changes also correlate directly with renal dysfunction, as shown by serum creatinine levels [9]. All these data suggest a role for gremlin in the pathogenesis of kidney damage.

Indeed, in the transgenic mice we found more acute tubular injury induced by folic acid than chronic damage progression. However it is important to note, than acute tubular injury is clearly associated with progression towards end stage renal disease [29], and on the other hand we found a significant interstitial cell infiltration that is commonly considered as the major initial mechanism leading to renal fibrosis.

Some evidence supports a potential interrelation between TGF-β1 and gremlin responses. We have previously demonstrated that *in vitro* blockade of endogenous gremlin by a specific siRNA inhibits TGF-β1-induced profibrotic gene overexpression and extracellular matrix production in renal fibroblasts. Moreover, gremlin blockade inhibits TGF-β1-mediated phenotypic-changes in tubular epithelial cells [12]. Even more, recently, it has been reported that gremlin likely induces endogenous TGF-β/Smad signaling, resulting in podocyte injury in mouse podocytes cultured in high glucose conditions [30]. Many data suggest that gremlin could be an important promoter of fibrosis in different pathologies, including liver fibrosis and lung diseases, particularly pulmonary hypertension, idiopathic pulmonary fibrosis and cancer invasion [31]-[35], as we have shown here in an experimental model of renal damage.

Our results suggest that GREM1-overexpressing mice have an increased susceptibility to renal damage, supporting the involvement of gremlin in renal damage progression.

## References

[1] Topol LZ, Marx M, Laugier D, Bogdanova NN, Boubnov NV, et al (1997) Identification of dmr, a novel gene whose expression is suppressed in transformed cells and which can inhibit growth of normal but not transformed cells in culture. Mol Cell Biol 17: 4801–4810. 9234736 10.1128/mcb.17.8.4801 PMC232332

[2] Topol LZ, Bardot B, Zhang Q, Resau J, Huillard E, et al (2000) Biosynthesis, post-translation modification, and functional characterization of Drm/Gremlin. J Biol Chem 275: 8785–8793. 10722723 10.1074/jbc.275.12.8785

[3] Hsu DR, Economides AN, Wang X, Eimon PM, Harland RM (1998) The Xenopus dorsalizing factor Gremlin identifies a novel family of secreted proteins that antagonize BMP activities. Mol Cell 1: 673–683. 9660951 10.1016/s1097-2765(00)80067-2

[4] Michos O, Panman L, Vintersten K, Beier K, Zeller R, et al (2004) Gremlin-mediated BMP antagonism induces the epithelial-mesenchymal feedback signaling controlling metanephric kidney and limb organogenesis. Development 131: 3401–3410. 15201225 10.1242/dev.01251

[5] Roxburgh SA, Murphy M, Pollock CA, Brazil D (2006) Recapitulation of embryological programmes in renal fibrosis–the importance of epithelial cell plasticity and developmental genes. Nephron Physiol 103: 139–148. 10.1159/000092453 16582577

[6] McMahon R, Murphy M, Clarkson M, Taal M, Mackensie H, et al (2000) IHG-2, a mesangial cell gene induced by high glucose, is human gremlin. Regulation by extracellular glucose concentration, cyclic mechanical strain, and transforming growth factor-beta1. J Biol Chem 275: 9901–9904. 10744662 10.1074/jbc.275.14.9901

[7] Murphy M, Godson C, Cannon S, Kato S, Mackenzie HS, et al (1999) Suppression subtractive hybridization identifies high glucose levels as a stimulus for expression of connective tissue growth factor and other genes in human mesangial cells. J Biol Chem 274: 5830–5834. 10026205 10.1074/jbc.274.9.5830

[8] Lappin DW, McMahon R, Murphy M, Brady HR (2002) Gremlin: an example of the re-emergence of developmental programmes in diabetic nephropathy. Nephrol Dial Transplant 17s9: 65–67. 10.1093/ndt/17.suppl_9.65 12386293

[9] Dolan V, Murphy M, Sadlier D, Lappin D, Doran P, et al (2005) Expression of gremlin, a bone morphogenetic protein antagonist, in human diabetic nephropathy. Am J Kidney Dis 45: 1034–1039. 15957132 10.1053/j.ajkd.2005.03.014

[10] Mezzano S, Droguett A, Burgos ME, Aros C, Ardiles L, et al (2007) Expression of gremlin, a bone morphogenetic protein antagonist, in glomerular crescents of pauci-immune glomerulonephritis. Nephrol Dial Transplant 22: 1882–1890. 17403698 10.1093/ndt/gfm145

[11] Carvajal G, Droguett A, Burgos ME, Aros C, Ardiles L, et al (2008) Gremlin: a novel mediator of epithelial mesenchymal transition and fibrosis in chronic allograft nephropathy. Transplant Proc 40: 734–739. 18455002 10.1016/j.transproceed.2008.02.064

[12] Rodríguez Diez R, Lavoz C, Carvajal G, Rayego-Mateos S, Rodríguez Diez RR, et al (2012) Gremlin is a downstream profibrotic mediator of transforming growth factor-beta in cultured renal cells. Nephron Exp Nephrol 122: 62–74. 23548835 10.1159/000346575

[13] Roxburgh SA, Kattla JJ, Curran SP, O′Meara YM, Pollock CA, et al (2009) Allelic depletion of grem1 attenuates diabetic kidney disease. Diabetes 58: 1641–1650. 19401426 10.2337/db08-1365 PMC2699858

[14] Zhang Q, Shi Y, Wada J, Malakauskas SM, Liu M, et al (2010) In vivo delivery of Gremlin siRNA plasmid reveals therapeutic potential against diabetic nephropathy by recovering bone morphogenetic protein-7. PLoS One 5: e11709. 20661431 10.1371/journal.pone.0011709 PMC2908623

[15] Farkas L, Farkas D, Gauldie J, Warburton D, Shi W, et al (2011) Transient overexpression of Gremlin results in epithelial activation and reversible fibrosis in rat lungs. Am J Respir Cell Mol Biol 44: 870–878. 20705941 10.1165/rcmb.2010-0070OC PMC3135847

[16] Ding Y, Davisson RL, Hardy DO, Zhu L, MerrilL DC, et al (1997) The kidney androgen-regulated protein promoter confers renal proximal tubule cell-specific and highly androgen-responsive expression on the human angiotensinogen gene in transgenic mice. J Biol Chem 272: 28142–28148. 9346970 10.1074/jbc.272.44.28142

[17] Ding Y, Sigmund C (2001) Androgen-dependent regulation of human angiotensinogen expression in KAP-hAGT transgenic mice. Am J Physiol Renal Physiol 280: F54–F60. 11133514 10.1152/ajprenal.2001.280.1.F54

[18] Lavoie JL, Lake-Bruse KD, Sigmund CD (2004) Increased blood pressure in transgenic mice expressing both human renin and angiotensinogen in the renal proximal tubule. Am J Physiol Renal Physiol 286: F965–F971. 15075192 10.1152/ajprenal.00402.2003

[19] Sachetelli S, Liu Q, Zhang SL, Liu F, Hsieh TJ, et al (2006) RAS blockade decreases blood pressure and proteinuria in transgenic mice overexpressing rat angiotensinogen gene in the kidney. Kidney Int 69: 1016–1023. 16528251 10.1038/sj.ki.5000210

[20] Li H, Zhou X, Davis DR, Xu D, Sigmund CD (2008) An androgen-inducible proximal tubule-specific Cre recombinase transgenic model. Am J Physiol Renal Physiol 294: F1481–F1486. 18385272 10.1152/ajprenal.00064.2008 PMC3584705

[21] Livak KJ, Schmittgen TD (2001) Analysis of relative gene expression data using real-time quantitative PCR and the 2(-Delta Delta C(T)) Method. Methods 25: 402–408. 11846609 10.1006/meth.2001.1262

[22] Zoja C, Corna D, Camozzi D, Cattaneo D, Rottoli D, et al (2002) How to fully protect the kidney in a severe model of progressive nephropathy: a multidrug approach. J Am Soc Nephrol 13: 2898–2908. 12444208 10.1097/01.asn.0000034912.55186.ec

[23] Ortega A, Rámila D, Ardura JA, Esteban V, Ruiz-Ortega M, et al (2006) Role of parathyroid hormone-related protein in tubulointerstitial apoptosis and fibrosis after folic acid-induced nephrotoxicity. J Am Soc Nephrol 17: 1594–1603. 16672315 10.1681/ASN.2005070690

[24] Phanish MK, Winn SK, Dockrell ME (2010) Connective tissue growth factor-(CTGF, CCN2)- a marker mediator and therapeutic target for renal fibrosis. Nephron Exp Nephrol 114: e83–92. 19955828 10.1159/000262316

[25] Yokoi H, Mukoyama M, Mori K, Kasahara M, Suganami T, et al (2008) Overexpression of connective tissue growth factor in podocytes worsens diabetic nephropathy in mice. Kidney Int 73: 446–455. 18075496 10.1038/sj.ki.5002722

[26] Alfaro MP, Deskins DL, Wallus M, DasGupta J, Davidson JM, et al (2013) A physiological role for connective tissue growth factor in early wound healing. Lab Invest 93: 81–95. 23212098 10.1038/labinvest.2012.162 PMC3720136

[27] Bonniaud P, Margetts PJ, Kolb M, Haberberger T, Kelly M, et al (2003) Adenoviral gene transfer of connective tissue growth factor in the lung induces transient fibrosis. Am J Respir Crit Care Med 168: 770–778. 12816739 10.1164/rccm.200210-1254OC

[28] Mori T, Kawara S, Shinozaki M, Hayashi N, Kakinuma T, et al (1999) Role and interaction of connective tissue growth factor with transforming growth factor-beta in persistent fibrosis: a mouse fibrosis model. J Cell Physiol 181: 153–159. 10457363 10.1002/(SICI)1097-4652(199910)181:1<153::AID-JCP16>3.0.CO;2-K

[29] Gentle ME, Shi S, Daehn I, Zhang T, Qi H, et al (2013) Epithelial cell TGF-β signaling induces acute tubular injury and interstitial inflammation. J Am Soc Nephrol 24: 787–799. 23539761 10.1681/ASN.2012101024 PMC3636798

[30] Li G, Li Y, Liu S, Shi Y, Chi Y, et al (2013) Gremlin aggravates hyperglycemia-induced podocyte injury by a TGFβ/Smad dependent signaling pathway. J Cell Biochem 114: 2101–2112. 23553804 10.1002/jcb.24559

[31] Guimei M, Baddour N, Elkaffash D, Abdou L, Taher Y (2012) Gremlin in the pathogenesis of hepatocellular carcinoma complicating chronic hepatitis C: an immunohistochemical and PCR study of human liver biopsies. BMC Res Notes 5: 390. 22839096 10.1186/1756-0500-5-390 PMC3506438

[32] Costello CM, Cahill E, Martin F, Gaine S, Mc Loughlin P (2010) Role of Gremlin in the lung: development and disease. Am J Respir Cell Mol Biol 42: 517–23. 19574532 10.1165/rcmb.2009-0101TR

[33] Cahill E, Costello CM, Rowan SC, Harkin S, Howell K, et al (2012) Gremlin plays a key role in the pathogenesis of pulmonary hypertension. Circulation 125: 920–930. 22247494 10.1161/CIRCULATIONAHA.111.038125

[34] Koli K, Myllärniemi M, Vuorinen K, Salmenkivi K, Ryynänen MJ, et al (2006) Bone morphogenetic protein-4 inhibitor gremlin is overexpressed in idiopathic pulmonary fibrosis. Am J Pathol 169: 61–71. 16816361 10.2353/ajpath.2006.051263 PMC1698771

[35] Karagiannis GS, Berk A, Dimitromanolakis A, Diamandis EP (2013) Enrichment map profiling of the cancer invasion front suggests regulation of colorectal cancer progression by the bone morphogenetic protein antagonist, gremlin-1. Mol Oncol 7: 826–839. 23659962 10.1016/j.molonc.2013.04.002 PMC5528431

