# Correction: Tubular Overexpression of Gremlin Induces Renal Damage Susceptibility in Mice

**DOI:** 10.1371/journal.pone.0112003

**Published:** 2014-10-22

**Authors:** The PLOS ONE Staff

There is an error in the Funding statement. Please refer to the complete funding statement here:

This study was supported by Ciberdem, Redinren, Fondecyt 1120480, Fondecyt 1080083, Fondecyt 1100821, Chile. The Centro de Estudios Científicos CECs is funded by the Centers of Excellence Base Financing Program of CONICYT. The funders had no role in study design, data collection and analysis, decision to publish, or preparation of the manuscript.

Additionally, there is an error in the sixth sentence of the “Transgenesis, genotyping and colony expansion” sub-heading in the Materials and Methods section. The correct sentence for this section is:

“Five hundred molecules were microinjected into hybrid C57BL/6J x CBA/J zygotes in the mouse facility of the Centro de Estudios Científicos-CECs, which were then transplanted into 13 pseudopregnant mothers.”

## References

[1] Droguett A, Krall P, Burgos ME, Valderrama G, Carpio D, et al (2014) Tubular Overexpression of Gremlin Induces Renal Damage Susceptibility in Mice. PLoS ONE 9(7): e101879 doi:10.1371/journal.pone.0101879 25036148 10.1371/journal.pone.0101879 PMC4103765

